# Outcomes after surgical correction of severe scoliosis in patients with osteogenesis imperfecta: a prospective, 2-year minimum follow-up study with radiographic and patient-reported outcomes

**DOI:** 10.1007/s43390-026-01340-y

**Published:** 2026-04-09

**Authors:** Satoshi Takada, Suken A. Shah, Hiroshi Taneichi, Yusuke Hori, Petya K. Yorgova, Andrea Elsby, Tyler C. McDonald, Richard W. Kruse, Jeanne M. Franzone

**Affiliations:** 1 Department of Orthopaedic Surgery, Nemours Children’s Health, 1600 Rockland Rd, Wilmington, DE 19803 USA; 2 Department of Orthopaedic Surgery, Dokkyo Medical University, Tochigi, Japan; 3 Department of Orthopaedic Surgery, Osaka City General Hospital, Osaka, Japan; 4 Department of Orthopaedic Surgery, University of South Alabama, Mobile, AL USA

**Keywords:** Scoliosis, Osteogenesis imperfecta, Posterior correction, Patient-reported outcomes

## Abstract

**Purpose:**

To evaluate both radiographic and patient-reported outcomes after surgical correction of severe scoliosis in osteogenesis imperfecta (OI) patients with minimum 2-year follow-up. The main research question was whether modern multimodal surgical approaches translate into meaningful improvements in both deformity correction and quality of life measures.

**Methods:**

This retrospective analysis of prospectively collected data included 35 OI patients (mean age 12.2 ± 3.7 years) who underwent posterior spinal fusion for scoliosis at a single institution between 2013 and 2022. OI type 3 was most common (69%). Radiographic parameters and patient-reported outcomes (SRS-22, CP CHILD, PROMIS) were assessed preoperatively and at latest follow-up (mean 5.5 ± 3.1 years). Surgical technique included navigation-guided pedicle screw placement, cement augmentation at foundation levels, and perioperative bisphosphonate therapy.

**Results:**

Major curve improved from 72.3 ± 15° to 32.2 ± 17° (56% correction, *p* < 0.001) and minor curve from 53.8 ± 14° to 24.1 ± 12° (55% correction, *p* < 0.001). Other radiographic parameters including apical vertebral translation, lowest instrumented vertebra tilt, pelvic obliquity, and thoracolumbar kyphosis showed significant improvement while maintaining thoracic kyphosis and lumbar lordosis. SRS-22 total score improved from 3.6 to 4.1 (*p* = 0.002), with function, pain, and self-image domains achieving minimal clinically important differences. CP CHILD total score improved, with significant improvement in the positioning/transferring/mobility domain. PROMIS showed significant improvements in fatigue and pain interference. Two complications (5.7%) occurred without infections or neurological deficits.

**Conclusion:**

Modern surgical correction of severe scoliosis in OI patients achieved significant improvements in both radiographic parameters and patient-reported outcomes, demonstrating the effectiveness of current multimodal surgical strategies in this challenging population.

## Introduction

Osteogenesis imperfecta (OI) is a rare genetic disorder of type I collagen, characterized by bone fragility and recurrent fractures [1, 2]. The majority of mutations (approximately 90%) are found in the COL1A1 and COL1A2 genes, which encode for type I collagen [3]. While multiple fractures and limb deformities are common clinical manifestations, spinal involvement represents one of the most challenging clinical manifestations in OI patients [3–5]. Although at least 17 genetic causes of OI have been identified, the phenotypic expression of the condition varies significantly in severity [6]. The prevalence of scoliosis in OI patients ranges from 18 to 80%, with higher rates observed in more severe phenotypes [7–10]. Spinal deformity in these patients can significantly impact quality of life through decreased physical function, restricted daily activities [11–13], and impaired respiratory function. These studies have shown that increasing severity of scoliosis correlates with a decrease in pulmonary function, specifically vital capacity [9, 14, 15].

The surgical management of scoliosis in OI patients presents challenges due to poor bone quality, increased bleeding tendency due to platelet dysfunction and vascular fragility [16], and high risk of complications. Traditional treatment methods, including bracing, have shown limited effectiveness and can be problematic due to fracture risk, fragility, and associated chest wall deformities. Historical surgical approaches including non-instrumented fusion and early instrumentation techniques have demonstrated modest curve correction and high complication rates up to 50% [17–20]. However, recent advances in surgical techniques and perioperative management have shown promising results [21, 22]. A contemporary multimodal approach integrating perioperative bisphosphonate therapy, preoperative/intraoperative traction, various osteotomies, segmental pedicle screw instrumentation with cement augmentation [23], and bone morphogenetic protein-2 (BMP-2) application has demonstrated improved outcomes with correction rates of 53% and minimal complications at mid-term follow-up [24].

Meanwhile, untreated scoliosis in these patients can lead to progressive deformity, decreased pulmonary function, and significantly impaired quality of life [9, 25]. Studies have shown that physical aspects of quality of life in OI patients with scoliosis appear to be lower than that of the general population, with restrictions in mobility and daily activities having substantial impact on their well-being [26]. However, there is limited evidence regarding how these improved surgical outcomes translate to patient-reported quality of life measures. Therefore, we conducted this study to evaluate both the radiographic and patient-reported outcomes after surgical correction of severe scoliosis in OI patients with a minimum 2-year follow-up.

## Methods

**Fig. 1:**
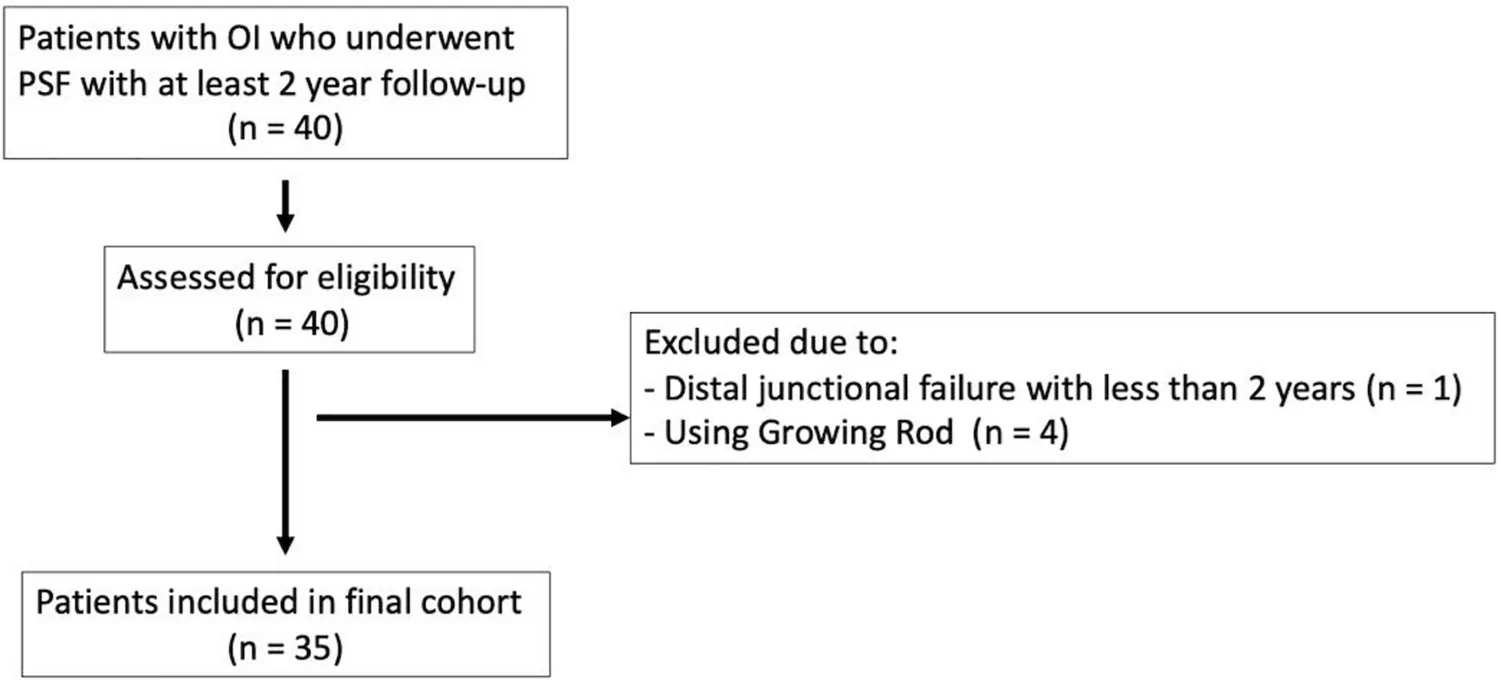
STROBE flow diagram of patient selection. Flow diagram showing the selection process for the study cohort. Of 40 patients with osteogenesis imperfecta (OI) who underwent posterior spinal fusion for scoliosis between 2013 and 2022, five patients were excluded: four due to treatment with growing rod instrumentation and one due to revision surgery for distal junctional kyphosis with less than 2 years of follow-up after the revision procedure. The final analysis included 35 patients who met inclusion criteria with minimum 2-year follow-up and complete radiographic and patient-reported outcome data

This single-center, single-surgeon retrospective analysis of prospectively collected data initially identified 40 patients with OI who underwent surgical treatment for scoliosis between 2013 and 2022. Inclusion criteria were as follows: (1) diagnosis of OI, (2) severe thoracolumbar scoliosis requiring posterior spinal fusion, and (3) radiographic and patient-reported outcome measures obtained at minimum 2-year follow-up after the initial surgery. Four patients were excluded due to treatment with growing rod instrumentation, and one patient was excluded due to having undergone revision surgery for distal junctional kyphosis with less than 2 years of follow-up after the revision. The remaining 35 patients were included in the final analysis (Fig. 1). Of the five patients with prior spinal surgery history, the procedures included two patients who had undergone cervical spinal fusion, one patient had undergone spinal fusion for fracture-related stabilization, and two patients had undergone single-level fusion. These patients were included in the analysis as their prior surgeries represented either unrelated procedures (cervical fusion) or limited procedures that would not significantly impact the outcomes of thoracolumbar deformity correction. The cohort had a mean age of 12.2 ± 3.7 years with equal sex distribution (50% male/female). Type III OI, the most severe form compatible with survival, was the most common phenotype, representing 69% of the cohort. The mean follow-up period was 5.5 ± 3.1 years. We also collected data on historical factors that could potentially impact quality of life outcomes, including previous fractures (including extremities) and prior spinal surgeries.

### Outcome measures

Clinical outcomes were assessed using both radiographic parameters and patient-reported outcome measures. Radiographic evaluation included major and minor curvature, apical vertebral translation (AVT), lowest instrumented vertebra (LIV) tilt, pelvic obliquity, coronal balance, thoracic kyphosis (T1–T12), thoracolumbar kyphosis (T10–L2), and lumbar lordosis. All measurements were performed on standard erect or sitting radiographs that were obtained preoperatively and at the latest follow-up. Curve classification was performed according to the Lenke classification system for structural and non-structural curves. Primary curves were identified as the largest structural curve, while secondary curves included compensatory curves above and below the primary curve. For patients with double major curves, both thoracic and thoracolumbar/lumbar curves were considered primary.

Patient-reported outcomes were evaluated using three validated instruments: the Scoliosis Research Society-22 questionnaire (SRS-22), the Caregiver Priorities and Child Health Index of Life with Disabilities (CP CHILD) [27], and the Patient-Reported Outcome Measurement Information System (PROMIS) Pediatric Form [28]. The SRS-22 domains include function, pain, self-image, mental health, and satisfaction. PROMIS measures included peer relationships, fatigue, pain interference, and physical function mobility. PROMIS scores were reported as T-scores, which have a mean of 50 and a standard deviation of 10 in the general population.

All patient-reported outcome measures were calculated by domain. The minimal clinically important difference (MCID) values that were established for adolescent idiopathic scoliosis surgery were used to determine the clinical significance of improvements in the SRS-22 domains. The MCID was 0.20 for the Pain domain, 0.08 for Function, and 0.98 for Appearance [29]. For CP CHILD, we utilized the MCID of 5.8 points for the total score established in cerebral palsy scoliosis patients, though specific MCID values for individual domains have not been established in the literature [30]. For PROMIS measures, we used previously reported MCID values in the literature: physical function (4.6), pain interference (4.0), and fatigue (4.0) [31–33].

### Surgical technique and perioperative management

All patients included in this review underwent posterior spinal fusion with segmental pedicle screw instrumentation. Preoperative examination included careful assessment of extremity range of motion and positioning considerations. Patient positioning in the operating room was performed by a specialized team experienced in caring for OI patients. Our surgical strategy incorporated several key elements to address the unique challenges of OI such as navigation-guided pedicle screw placement, apical posterior column osteotomies (PCO), rib osteotomies (when necessary), cement augmentation at the proximal and distal foundation levels, and local autograft combined with bone morphogenetic protein-2 for fusion. Traction strategies were employed based on curve severity, rigidity, and patient-specific factors rather than strict angular criteria. Preoperative halo-gravity traction was utilized in selected cases with rigid, severe curves, typically initiated 2–4 weeks before definitive surgery with gradual weight increase as tolerated. Intraoperative traction was applied in the majority of cases to facilitate deformity correction and protect neural elements during instrumentation. The decision to use preoperative versus intraoperative traction alone was made on a case-by-case basis, considering curve rigidity assessed on bending radiographs, overall curve severity, and patient tolerance. Pelvic fixation was performed in cases with significant pelvic obliquity; however, we generally avoided fusion to the pelvis whenever possible due to associated acetabular protrusio, poor hip motion, and limitations of pelvic fixation related to narrowing of inner and outer tables with minimal cortical bone. Special consideration was also given to the challenges posed by severe lumbar lordosis common in these patients. Multimodal intraoperative and postoperative pain management protocols were implemented for all patients. All patients received a standardized perioperative pamidronate protocol consisting of 1 mg/kg/day for 3 consecutive days, administered 1 to 4 weeks preoperatively. No variations in the protocol occurred based on age alone, though the weight-based calculation naturally adjusted doses for smaller pediatric patients. Bisphosphonate administration was then suspended for 4 to 6 months postoperatively until radiographic evidence of fusion mass formation was confirmed. Additionally, optimization of Vitamin D levels and calcium intake was ensured. Bisphosphonate administration was then suspended for 4 to 6 months postoperatively until fusion mass was confirmed. Intraoperative spinal cord monitoring was performed in all cases. The multimodal approach described in this study was implemented systematically at our institution beginning in 2013, coinciding with the study period. Prior to 2013, our center used conventional instrumentation without routine cement augmentation or navigation guidance, and bisphosphonate protocols were less standardized. All patients included in this study received the contemporary multimodal treatment approach.

### Statistical analysis

Continuous variables were presented as mean with standard deviation. Data normality was assessed using the Shapiro–Wilk test. For normally distributed data, changes in radiographic parameters and patient-reported outcomes between preoperative and final follow-up measurements were analyzed using paired t-tests. For non-normally distributed data, the Wilcoxon signed-rank test was used. A *p* value less than 0.05 was considered statistically significant. All analyses were performed using JMP Pro version 18 (SAS Institute Inc., Cary, NC).

## Results

### Patient characteristics

The study cohort consisted of 35 patients with a mean age of 12.2 ± 3.7 years at the time of surgery, with equal sex distribution (50% male/female). Type III OI was the most common phenotype (69%), followed by type IV (20%), type I (7%), and others (4%) (Table 1). The mean follow-up period was 5.5 ± 3.1 years. Curve patterns included single thoracic curves in 22 patients (65%), single thoracolumbar/lumbar curves in five patients (14%), and double major curves in eight patients (23%). For patients with double major curves, the mean thoracic curve was 68.2 ± 12° and thoracolumbar curve was 64.8 ± 18° preoperatively. Prior spinal surgery history was as follows: 30 patients had no prior surgeries, two patients had one prior surgery, two patients had two prior surgeries, and one patient had more than three surgeries. Regarding fracture history, one patient had one to five fractures, two patients had six to 11 fractures, two patients had 12–20 fractures, and 30 patients had more than 20 or uncountable fractures. The number of prior orthopedic surgeries was distributed as follows: eight patients had one to five surgeries, 10 patients had six to 10 surgeries, and 17 patients had more than 10 or uncountable surgeries (Table 2).

**Table 1:** Demographic and clinical characteristics of patients with osteogenesis imperfecta

	Mean (SD) or *n* (%)
Age, y	12.2 (3.7)
Sex (Female)	17 (49.6%)
BMI	19.9 (6.0)
OI type	
Type 1	2 ( 5%)
Type 2	1 (3%)
Type 3	24 (69%)
Type 4	5 ( 14%)
Type 5	2 ( 5%)
Type 8	1( 3%)
Curve type	
Single thoracic	22 (65%)
Single thoracolumbar/lumbar	5 (14%)
Double major	8 (23%)
Major curvature, °	72.3(15.1)
Follow-up period, years	5.5 (3.1)

**Table 2:** Prior surgical history and fracture profiles of the study cohort

	*n* (%)
Prior spinal surgery history	
No prior surgeries	30 (85.7%)
One prior surgeries	2 (5.7%)
Two prior surgeries	2 (5.7%)
More than three surgeries	1 (2.9%)
Fracture history	
1–5 fractures	1 (2.9%)
6–11 fractures	2 (5.7%)
12–20 fractures	2 (5.7%)
More than 20 or uncountable fractures	30 (85.7%)
Prior orthopedic surgeries	
1–5 surgeries	8 (24.4%)
6–10 surgeries	10 (31.1%)
More than 10 or uncountable surgeries	17 (44.5%)

### Radiographic outcomes

Significant improvements were observed in all major coronal plane parameters at final follow-up. The major curvature improved from 72.3 ± 15 (range 112–52)° preoperatively to 32.2 ± 17° at final follow-up (*p* < 0.001), representing a 56% correction. The minor curvature showed similar improvement from 53.8 ± 14 (range 34–80)° to 24.1 ± 12° (*p* < 0.001), a 55% correction. Significant improvements were also seen in other coronal parameters: AVT decreased from 53.9 ± 21 mm to 18.8 ± 19 mm (*p* < 0.001), LIV tilt improved from 25.9 ± 10° to 10.2 ± 7° (*p* < 0.001), and pelvic obliquity decreased from 9.9 ± 6° to 6.9 ± 1° (*p* = 0.04). In the sagittal plane, while T1–T12 kyphosis (30.1 ± 28° to 34.1 ± 18°, *p* = 0.56) and lumbar lordosis (52.6 ± 28° to 55.2 ± 19°, *p* = 0.34) remained stable, thoracolumbar kyphosis (T10-L2) showed significant improvement from 25.9 ± 24° to 8.3 ± 11° (*p* < 0.001) (Table 3).

**Table 3:** Comparison of radiographic parameters between preoperative and final follow-up

Parameters	Pre-op (Mean ± SD)	Follow-up (Mean ± SD)	*p* value
Major curvature, °	72.3 ± 15	32.2 ± 17	< 0.001^*^
Minor curvature, °	53.8 ± 14	24.1 ± 12	< 0.001^*^
AVT, mm	53.9 ± 21	18.8 ± 19	< 0.001^*^
LIV tilt, °	25.9 ± 10	10.2 ± 7	< 0.001^*^
Pelvic obliquity, °	9.9 ± 6	6.9 ± 1	0.04^*^
Coronal balance, mm	22.3 ± 20	8.3 ± 29	0.57
T1–T12 kyphosis, °	30.1 ± 28	34.1 ± 18	0.56
T10–L2 kyphosis, °	25.9 ± 24	8.3 ± 11	< 0.001^*^
Lumbar lordosis, °	52.6 ± 28	55.2 ± 19	0.34

^*^p < 0.05 compared with preoperative

### Surgical details

The mean number of levels fused was 13.3 ± 2.0 levels. Upper instrumented vertebra (UIV) distribution was as follows: C7 in one patient (3%), T1 in four patients (10%), T2 in seven patients (20%), T3 in 19 patients (53%), and T4 in four patients (13%). Lower instrumented vertebra (LIV) distribution included T12 in two patients (7%), L1 in four patients (10%), L2 in six patients (17%), L3 in 14 patients (40%), L4 in six patients (17%), and L5 in one patient (3%). Pelvic fixation was performed in two patients (7%). All patients received segmental pedicle screw instrumentation with cement augmentation at the proximal and distal foundation levels. The average number of pedicle screws per patient was 18.3 ± 4.2. Our instrumentation strategy involved bilateral screw placement at the proximal and distal two to three levels to create strong foundation points, with selective screw placement at intermediate levels to balance stability and minimize implant burden. Regarding traction strategies, three patients (9%) underwent preoperative halo-gravity traction for 2–4 weeks prior to definitive surgery, 28 patients (80%) received intraoperative traction only, and four patients (11%) underwent surgery without traction. The mean estimated blood loss was 755 ± 510 ml, and the mean length of hospital stay was 5.4 ± 2.3 days (Table 4).

**Table 4:** Surgical details

	Mean ± SD or *n* (%)
Number of fused vertebrae	13.3 ± 2.0
Upper instrumented vertebra (UIV)	
C7	1 (2.9%)
T1	4 (11.4%)
T2	7 (20.0%)
T3	19 (54.3%)
T4	4 (11.4%)
Lower instrumented vertebra (LIV)	
T12	2 (5.7%)
L1	4 (11.4%)
L2	6 (17.1%)
L3	14 (40.0%)
L4	6 (17.1%)
L5	1 (2.9%)
Pelvic fixation	2 (5.7%)
Traction strategy	
Preoperative halo-gravity traction	3 (8.6%)
Intraoperative traction only	28 (80.0%)
No traction	4 (11.4%)
Average number of pedicle screws	18.3 ± 4.2
Estimated blood loss, ml	755 ± 510
Length of hospital stay, days	5.4 ± 2.3

### Patient-reported outcomes

Significant improvements were observed across multiple patient-reported outcome measures. The SRS-22 total score improved from 3.8 to 4.2 (*p* = 0.002). Individual SRS-22 domains showed significant improvements as follows: function improved from 3.6 to 4.1 (*p* = 0.002), pain from 3.8 to 4.3 (*p* = 0.009), and self-image from 3.5 to 4.2 (*p* = 0.001), all exceeding their respective MCID values. The satisfaction domain also showed significant improvement from 3.7 to 4.6 (*p* < 0.001). The CP CHILD total score improved from 73.1 to 80.2 (*p* = 0.01), with significant improvement in the positioning/transferring/mobility domain (69.9 to 73.6, *p* = 0.04). PROMIS measures demonstrated significant improvements in several domains: fatigue scores improved from 14.2 to 8.0 (*p* = 0.01) and pain interference decreased from 15.4 to 8.7 (*p* = 0.02), both exceeding the established MCID thresholds. The 95% confidence intervals (CI) for key outcome improvements were SRS-22 total score change 0.46 (95% CI 0.42–0.50), SRS-22 self-image domain change 0.74 (95% CI 0.68–0.80), PROMIS fatigue improvement 6.2 (95% CI 5.40–7.00), and PROMIS pain interference improvement 6.7 (95% CI 5.72–7.68). Effect sizes for the significant improvements were very large: fatigue domain (Cohen’s *d* = 2.67) and pain interference domain (Cohen’s *d* = 2.34), indicating clinically meaningful changes beyond statistical significance. When stratified by major OI types, both type III (*n* = 24) and type IV (*n* = 5) patients showed similar patterns of improvement in SRS-22 and PROMIS domains, though formal statistical comparison was not performed due to small subgroup sizes (Table 5).

**Table 5:** Changes in patient-reported outcome measures between preoperative and final follow-up

Patient-reported outcomes: Mean(SD)	Pre-op	Post-op	*p* value
SRS-22 total score	3.75 (0.08)	4.22 (0.08)	0.002^*^
SRS-22 general function	3.60 (0.52)	4.06 (0.52)	0.002^*^
SRS-22 mental health	4.12 (0.13)	4.17 (0.12)	0.78
SRS-22 pain	3.81 (0.12)	4.25 (0.11)	0.009^*^
SRS-22 self-image	3.49 (0.67)	4.23 (0.61)	0.001^*^
SRS-22 satisfaction	3.69 (0.87)	4.63 (0.57)	< 0.001^*^
CPCHILD total score	73.1 (2.1)	80.2 (1.8)	0.01^*^
CPCHILD activities of daily living	85.9 (4.0)	84.3 (3.4)	0.77
CPCHILD positioning, transferring, mobility	69.9 (4.4)	73.6 (3.7)	0.04^*^
CPCHILD comfort and emotions	70.3 (4.3)	69.8 (3.0)	0.92
CPCHILD communication and social interaction	77.2 (3.5)	80.5 (3.0)	0.48
CPCHILD health	72.0 (5.4)	77.9 (4.6)	0.41
PROMIS pediatric peer relationships	24.2 (1.7)	28.0 (1.4)	0.08
PROMIS pediatric fatigue	14.2 (1.8)	8.0 (1.5)	0.01^*^
PROMIS pediatric pain interference	15.4 (2.2)	8.7 (1.8)	0.02^*^
PROMIS pediatric physical function: mobility	14.6 (1.8)	15.8 (1.5)	0.61

^*^p < 0.05 compared with preoperative

### Complications

The overall complication rate was low, with two major complications observed during the study period. One patient developed proximal junctional kyphosis (PJK) at 18 months postoperatively and subsequently underwent proximal extension surgery. This patient was included in the analysis as adequate 2-year follow-up was achieved after the revision procedure. Another patient sustained a femoral fracture 1-month postoperatively but was included in the analysis as 2-year follow-up was completed from the initial spinal surgery. No surgical site infections or neurological deficits were observed during the follow-up period. Additionally, no rod breakage or significant correction loss was observed during our follow-up period, though continued surveillance for these potential late complications remains important given the unique challenges of maintaining stable constructs in OI patients.

**Fig. 2:**
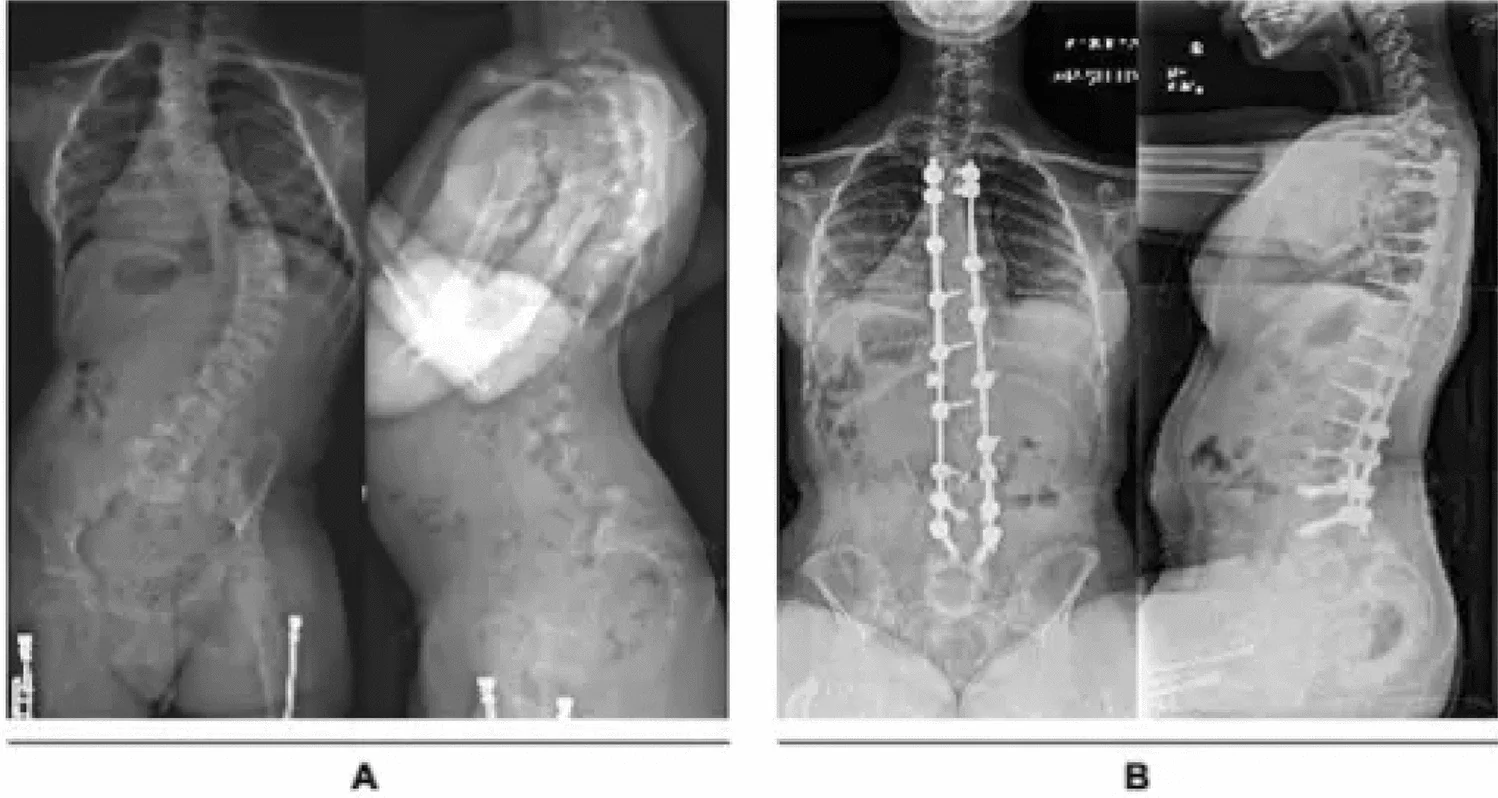
**A** Preoperative radiographs of a 15-year-old female with type III osteogenesis imperfecta (OI) showing curvatures of 75° and 55°. Preoperative measurements included apical vertebral translation (AVT) of 77 mm, thoracic kyphosis of 22°, thoracolumbar kyphosis of 33°, and lumbar lordosis of 75°. Preoperative SRS-22 total score was 3.2 (pain 4.0, function 3.0, self-image 2.2, mental health 3.4, satisfaction 4.0), CP CHILD total score was 60 (activities of daily living 100, positioning/transferring/mobility 69, comfort and emotions 57.5, communication and social interaction 65, health 60), and PROMIS T-scores were peer relationships 10, fatigue 15, pain interference 18, and physical function mobility 18. **B** 2-year postoperative radiographs following posterior spinal fusion from T3 to L4 with segmental pedicle screw instrumentation and cement augmentation at the proximal (T3, T4) and distal (L2, L3, L4) foundation levels. Radiographic measurements showed correction of major curves to 11° and 12° with AVT of 14 mm. Sagittal parameters were corrected to thoracic kyphosis of 30°, thoracolumbar kyphosis of 22°, and lumbar lordosis of 57°. Patient-reported outcomes improved with SRS-22 total score of 4.1 (pain 4.2, function 4.0, self-image 4.4, mental health 4.0, satisfaction 4.0), CP CHILD total score of 65 (activities of daily living 100, positioning/transferring/mobility 69, comfort and emotions 87, communication and social interaction 68, health 90), and PROMIS T-scores of peer relationships 14, fatigue 9, pain interference 13, and physical function mobility 14

**Fig. 3:**
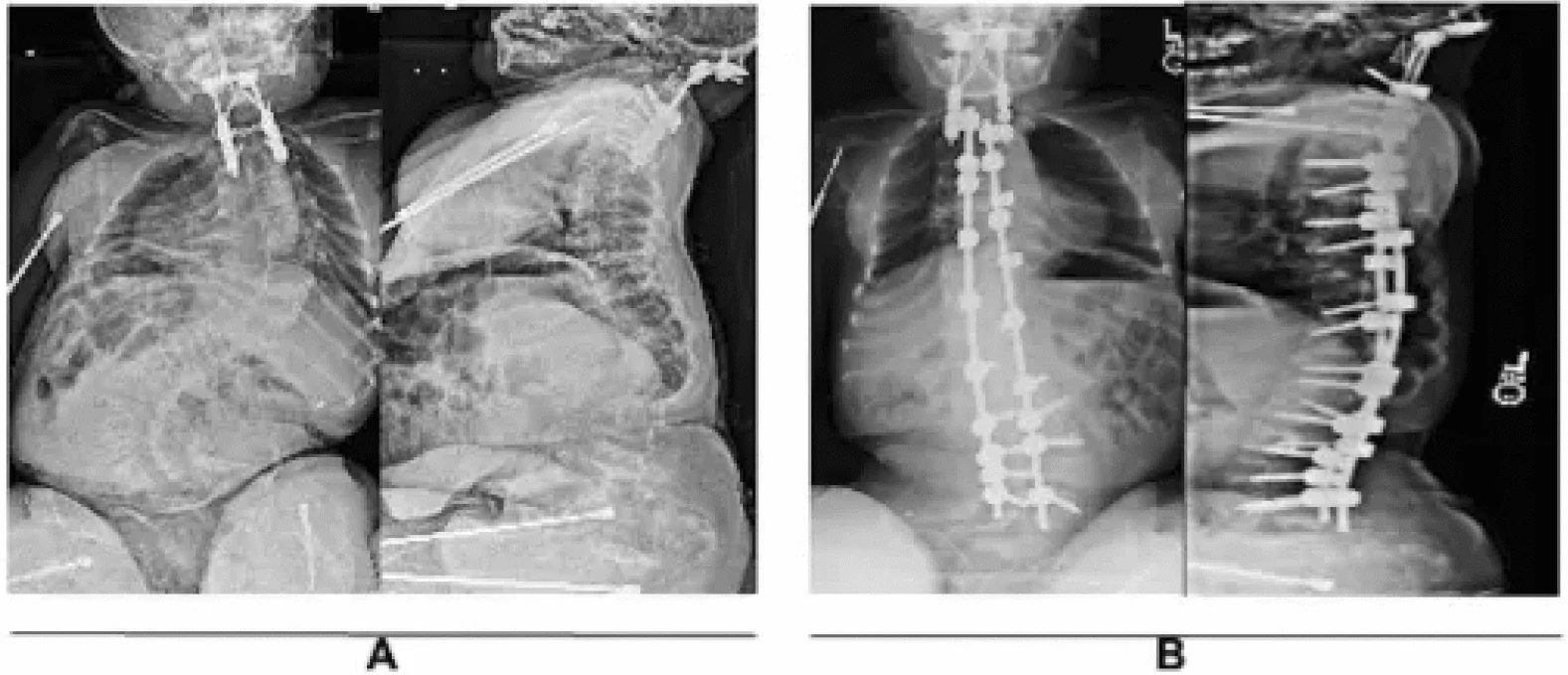
**A** Preoperative radiographs of a 14-year-old female with type IV osteogenesis imperfecta (OI) and history of prior cervical spinal fusion showing double major curvatures of 112° and 75°. Preoperative measurements included apical vertebral translation (AVT) of 92 mm, thoracic kyphosis of − 38°, thoracolumbar kyphosis of 31°, and lumbar lordosis of 75°. Preoperative SRS-22 total score was 3.0 (pain 2.8, function 3.3, self-image 2.4, mental health 3.4, satisfaction 3.0), CP CHILD total score was 52 (activities of daily living 52, positioning/transferring/mobility 45, comfort and emotions 40, communication and social interaction 55, health 50), and PROMIS T-scores were peer relationships 12, fatigue 25, pain interference 28, and physical function mobility 15. **B** 2-year postoperative radiographs following posterior spinal fusion with segmental pedicle screw instrumentation. The proximal construct was connected to the existing cervical hardware, while cement augmentation was performed at the distal foundation levels. Radiographic measurements showed correction of major and minor curves to 31° and 28°, respectively, with AVT of 23 mm. Sagittal parameters were corrected to thoracic kyphosis of 40°, thoracolumbar kyphosis of 2°, and lumbar lordosis of 52°. Patient-reported outcomes improved with SRS-22 total score of 3.7 (pain 3.6, function 4.0, self-image 3.4, mental health 3.6, satisfaction 4.5), CP CHILD total score of 67 (activities of daily living 74, positioning/transferring/mobility 58, comfort and emotions 53, communication and social interaction 68, health 80), and PROMIS T-scores of peer relationships 16, fatigue 20, pain interference 17, and physical function mobility 22

Two representative cases are presented to illustrate the range of corrections achievable with our multimodal surgical approach. Figure 2 demonstrates a 15-year-old female with type III OI who presented with a single thoracic curve and underwent posterior spinal fusion. Figure 3 shows a 14-year-old patient with type IV OI and a history of prior cervical spinal fusion who underwent surgical correction of a double major curve pattern. Both cases exemplify the substantial radiographic improvements and maintenance of sagittal balance observed across different curve types, patient ages, and complexity levels in our series, including patients with previous spinal surgery history. Representative pre- and postoperative radiographs demonstrate not only significant curve correction but also corresponding improvements in patient-reported quality of life outcomes.

## Discussion

This study demonstrates the effectiveness of a multimodal surgical approach for treating severe scoliosis in OI patients, focusing on both radiographic and patient-centered outcomes. In addition to achieving substantial deformity correction with a low complication rate, we also found that surgical intervention led to meaningful improvements in patients’ quality of life. Notably, improvements in multiple patient-reported outcome measures exceeded established MCID thresholds, suggesting that radiographic correction translated into tangible quality of life benefits for patients. While previous studies have focused primarily on radiographic outcomes or surgical complications, our study uniquely combines comprehensive radiographic analysis with validated patient-reported outcome measures (SRS-22, CP CHILD, and PROMIS) in OI patients undergoing modern multimodal surgical correction. To our knowledge, this represents the largest series specifically evaluating patient-reported outcomes in OI scoliosis surgery using contemporary techniques including navigation-guided pedicle screw instrumentation with cement augmentation and perioperative bisphosphonate optimization. Furthermore, our demonstration that improvements in multiple patient-reported domains exceed established minimal clinically important differences provides crucial evidence that radiographic correction translates into meaningful quality of life benefits for these patients—a relationship that has not been well-established in the OI population previously.

The radiographic outcomes in our series demonstrate significant improvement compared with historical reports of surgical treatment for scoliosis in OI patients. While early surgical series using traditional instrumentation techniques reported limited correction rates of 30–40% with complication rates and unplanned reoperations of up to 50%, contemporary studies using pedicle screw fixation have shown improved outcomes [17, 20]. Recent series have reported correction rates ranging from 48 to 59%, though some noted significant correction loss over time [22]. At latest follow-up, our curve correction rate of 56% was achieved through our multimodal approach, encompassing perioperative bisphosphonate administration, preoperative/intraoperative traction, osteotomy to alleviate rigid curves, segmental pedicle screw fixation with cement augmentation, and BMP-2 application [23, 24]. The stability of thoracic kyphosis and improvement of thoracolumbar kyphosis in our series suggest that appropriate sagittal alignment can be achieved while maintaining substantial coronal correction, addressing historical concerns about sagittal plane decompensation in this patient population [34].

Beyond radiographic improvements, this study demonstrates significant enhancement in patient-reported outcomes across multiple domains. This is particularly meaningful as untreated scoliosis in OI patients typically leads to progressive functional decline and decreased quality of life over time [9, 25], creating a widening gap between treated and untreated patients. The improvements in SRS-22 scores for function (3.6 to 4.1), pain (3.8 to 4.3), and self-image (3.5 to 4.2) domains not only achieved statistical significance but also exceeded their respective MCID thresholds of 0.08, 0.20, and 0.98 [29]. The substantial improvement in the self-image domain was particularly noteworthy as this improvement reflects the psychological impact of correcting severe spinal deformity in this population. Furthermore, significant improvements in PROMIS measures were observed in both fatigue (14.2 to 8.0) and pain interference (15.4 to 8.7) domains, exceeding the established MCID of 4.0 for each domain [31–33]. These improvements are particularly important as both fatigue and pain are reported in the OI literature to be significant issues affecting quality of life in OI patients [3]. The baseline PROMIS scores in our cohort were indeed lower than population norms, reflecting the significant functional limitations experienced by OI patients with severe spinal deformity. The very large effect sizes (*d* > 2.0) for both fatigue and pain interference domains demonstrate that surgical correction resulted in substantial improvements that are both statistically and clinically meaningful. The improvement in CP CHILD positioning/transferring/mobility domain scores also indicates enhanced functional capabilities, which is particularly relevant for OI patients who often face mobility challenges [35, 36]. Additionally, our CP CHILD total score improvement of 7.1 points exceeded the established MCID of 5.8 points, confirming the clinical significance of these functional gains.

The interpretation of patient-reported outcomes in OI patients requires careful consideration of their complex medical histories. In our cohort, more than 85% of patients had experienced either more than 20 fractures or had uncountable fracture episodes, while nearly 45% had undergone more than 10 orthopedic procedures prior to their spinal surgery. This extensive history of fractures and surgeries could potentially influence patient-reported outcome measures, as patients may have developed different baseline expectations and adaptations to physical limitations. Despite these challenging backgrounds, the consistent improvements across multiple patient-reported domains suggest the positive impact of successful spinal deformity correction in this population. The two major complications in our series—one case of PJK requiring revision and one postoperative femoral fracture—did not appear to significantly impact the overall positive trends in patient-reported outcomes. This observation suggests that when complications are recognized early and managed appropriately, patients can still achieve satisfactory clinical outcomes.

This study has several important limitations. First, despite representing the largest reported series of OI patients undergoing modern pedicle screw fixation with cement augmentation, our sample size of 35 patients limits our ability to perform meaningful analyses based on genotype–phenotype correlations. This would be valuable for tailoring surgical strategies to specific OI types. While our preliminary observations suggest similar improvement patterns across different OI types, formal statistical comparison was not feasible given the small numbers in each subgroup. Second, while we demonstrated significant improvements in patient-reported outcomes, these measures can be influenced by factors beyond surgical intervention, such as ongoing medical management, caregiver bias, physical therapy, and natural adaptation to physical limitations. Furthermore, we did not perform correlation analysis between patient-reported outcome scores and specific radiographic parameters, limiting our ability to establish direct relationships between the magnitude of deformity correction and quality of life improvements. Additionally, the MCID thresholds we used were derived from adolescent idiopathic scoliosis or general pediatric populations rather than OI-specific cohorts. The applicability of these MCID values to OI patients remains uncertain given the distinct pathophysiology, baseline functional limitations, and adaptation mechanisms in this population. OI-specific MCID thresholds have not been established in the literature, representing an important area for future research. Finally, while our mean follow-up of 5.5 years provides valuable mid-term data, longer surveillance is needed to assess potential late complications including rod breakage, progressive proximal junctional kyphosis, and correction loss in this poor bone quality population. Formal Kaplan–Meier survival analysis for implant-related complications would be valuable in future studies with larger cohorts and extended follow-up. We acknowledge that the inclusion of patients with prior spinal surgery history may represent a potential bias in both radiographic and patient-reported outcomes. However, sensitivity analysis excluding these five patients showed similar trends in outcome improvements, suggesting that prior surgeries did not significantly confound our main findings. A limitation of our study is the lack of a control group treated with conventional techniques for direct comparison. While historical controls treated before 2013 using traditional methods showed higher complication rates and more modest curve corrections in our institutional experience, formal statistical comparison was not feasible due to differences in patient selection criteria and outcome measurement protocols over time. Additionally, as a single-surgeon, single-institution study, our results may not be generalizable to other centers with varying surgical expertise, resources, or experience in treating this rare condition. The multimodal approach requires specialized equipment and technical expertise that may not be available at all institutions treating OI patients.

## Conclusion

In conclusion, our study demonstrates that a multimodal surgical approach for severe scoliosis in OI patients can achieve significant improvements in both radiographic parameters and patient-reported outcomes at mid-term follow-up. Our strategy of incorporating perioperative bisphosphonate therapy, navigation-guided pedicle screw placement with selective cement augmentation, and careful surgical planning resulted in a satisfactory correction rate with a low complication profile. The improvements in patient-reported outcome measures, particularly in domains related to self-image, pain, and function, suggest that successful deformity correction positively impacts quality of life in these patients. Future studies with larger cohorts may help further refine surgical strategies based on specific OI types and establish optimal protocols for perioperative management in this challenging patient population. With improvement in operative care of OI patients with spinal deformities, patients should be counseled to seek care early in the evolution of the condition so that meaningful care can be delivered, and the outcomes can be more impactful.
